# Long-term effects of combination of organic and inorganic fertilizer on soil properties and microorganisms in a Quaternary Red Clay

**DOI:** 10.1371/journal.pone.0261387

**Published:** 2021-12-16

**Authors:** Yiren Liu, Zhenzhen Lv, Hongqian Hou, Xianjin Lan, Jianhua Ji, Xiumei Liu

**Affiliations:** 1 Institute of Soil Fertilizer and Resource Environment, Jiangxi Academy of Agricultural Sciences, Nanchang, China; 2 National Engineering and Technology Research Center for Red Soil Improvement, Nanchang, China; COMSATS University Islamabad - Abbottabad Campus, PAKISTAN

## Abstract

Quaternary Red Clay (QRC) is the most common planting soil with low soil fertility and low crop yields in Southeast China, with low soil fertility and low crop yields. Many factors can impact the fertility and utilization efficiency of QRC. Here, we conducted a long-term fertilization experiment from 1984 to 2013. Five fertilization measures were carried out, including non-fertilization group; chemical Fertilizer group; 70% chemical and 30% organic fertilizer group; 50% chemical and 50% organic fertilizer group; 30% chemical and 70% organic fertilizer group. Soil organic matter (OM), total nitrogen (TN), total phosphorus (TP), total potassium (TK), soil microbial biomass carbon (SMBC) and nitrogen (SMBN), and soil enzymes activity were measured to evaluate the changes of soil. In addition, soil microorganisms were determined by high-throughput sequencing technology, and the dominant microbes were screened. The higher the proportion of organic fertilizer was, the higher the soil OM content was. The OM content of the non-fertilization group was the lowest. Similarly, SMBC and SMBN showed a consistent trend with OM content. Illumina sequence results showed that the application of organic fertilizer reduced the relative abundance of *Chloroflexi*, *Acidobacteria* and *Nitrospirae*, but increased *Proteobacteria* and *Actinobacteria*. The relative abundance of *Acremonium* and *Mortierella* were also greatly increased by different fertilization strategies. However, when high proportion of organic fertilizer was applied, the abundance of *Acremonium* and *Mortierella* decreased. Long-term balanced inorganic fertilization (NPK, 60%N:20%P:20%K) can effectively improve the quality and fertility of QRC. The effect of different fertilization strategies on fungi was greater than that on bacteria. The change of soil microorganism also proved the validity of inorganic fertilizer application.

## Introduction

Soil organic matter is one of the important evaluation indexes of soil quality. Generally, the application of organic fertilizer could improve the soil structure and the content of soil organic matter, which then improve the yield and quality of crops [1]. Quaternary Red Clay (QRC) is the most common planting soil in Southeast China, with low soil fertility and low crop yields. Lots of work had been carried out to improve QRC fertility, and some progresses had been made [2–4]. But a lot of work is still needed to improve the utilization efficiency of QRC.

The content of soil organic matter can be influenced by many ways, such as fertilization or cultivation of land [5, 6], of which fertilization is an important agricultural practice for improving nutrition of plants, reaching high yield, and changing important components of soil, such as the chemistry of soil carbon and nitrogen [7]. Healthy soils are usually rich in soil microorganisms and high in enzyme activity, and gradually formed a virtuous circle [8]. Soil microorganisms, the important components of microbial biomass, are vital to agroecosystem health through their roles in residue decomposition, nutrient cycling and their associations with other organisms [9]. Chu reported that organic fertilizers usually increase soil microbial biomass, carbon dioxide evolution and enzyme activities, whereas inorganic fertilizers have relatively less effect on these soil properties [10]. Organic fertilizers could greatly change the activity and diversity of soil microorganisms [11, 12]. Therefore, microorganisms act as indicators of soil environment condition changing and microbial biomass quantity [13]. Godara *et al*. believed that the best remedy for soil fertility management is where the inorganic fertilizer provides nutrients and the organic fertilizer mainly increases soil organic matter and improves soil structure and buffering capacity of the soil [14], which provided new strategies for the improvement of planting soil with low fertility. Our knowledge of microbial diversity in QRC is extreme exhaustion and the effect of organic fertilizer application on QRC is also unclear. In order to improve the soil fertility of QRC, we carried out different fertilization strategies for more than 30 years to verify the quality improvement effects on QRC.

In this study, we focus on the changes of microbial diversity in QRC paddy soil after long-term different fertilization strategies treatment, including chemical fertilizer and combination of organic and inorganic fertilizer in different proportion. The next generation sequencing was used to study microbial community and diversity. These results will show the long-term effects of different fertilization strategies on QRC soil structure and microbial composition and provide new insights for QRC improvement.

## Materials and methods

### Experimental design, location and sampling

In order to study the long-term effects of different organic fertilization strategies on Quaternary Red Clay (QRC), different organic fertilization strategies were performed since 1984. The quaternary red soil experimental sites of Jiangxi Academy of Agricultural Sciences were located in Nanchang County (E115°93’, N28°55’), Nanchang City, Jiangxi Province. The basic climate of the test sites is as follows: Annual Average Temperature (AAT): ~17.5°C; Effective Accumulated Temperature (EAT): 5400°C; Average Annual Precipitation (AAP): 1600 mm; Mean Annual Evaporation (MAE): 1800 mm; Frost-Free Period (FFP): ~280 d.

**Fig 1:**
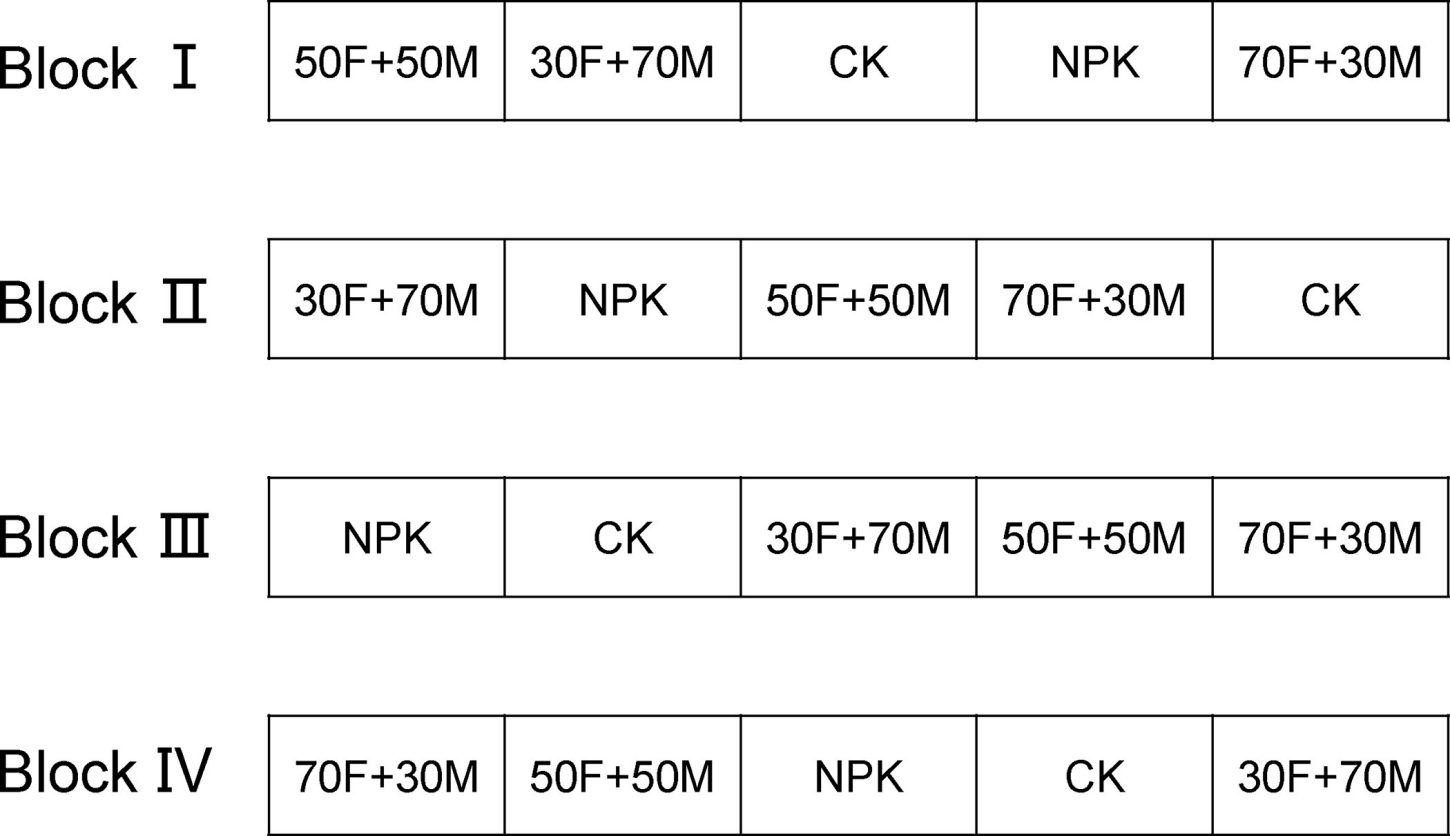
Schematic diagram of planting zoning. Each plot was 33.3 m^2^ with 0.5m × 0.5m cement ridges around the block. Each block as a repeat. CK: non-fertilization group; NPK: balanced inorganic fertilization; 70F+30M: 70% chemical and 30% organic fertilizer group; 50F+50M: 50% chemical and 50% organic fertilizer group; 30F+70M: 30% chemical and 70% organic fertilizer group.

A total of five groups were set up for different fertilization strategies: non-fertilization group (CK); balanced inorganic fertilizer group (NPK, 60%N:20%P:20%K); 70% chemical and 30% organic fertilizer group (70F+30M); 50% chemical and 50% organic fertilizer group (50F+50M); 30% chemical and 70% organic fertilizer group (30F+70M). For each group, four randomized and independent field plots were designed as replications. The design of this study was completely randomized block design. The area of each plot was 33.3 m^2^ with 0.5m × 0.5m cement ridges around the block. The experimental fields were used for rice planting, and a double-season cropping system were conducted using early- and late-season rice varieties. All test plots are consistent except for different fertilization strategies (Fig 1).

Surface paddy soil samples (0–20 cm) were collected from the 20 sampling field plots after the rice harvest. Soil samples were collected every five years (science 1983). All the collected samples were dried indoors, crushed with a laboratory mill (TAISETE, Tianjin, China) and sieved in 1mm Soil Analysis Sieves (SAS). All the pretreated soil samples were stored at -80°C. All soil samples were used for soil physicochemical properties detection. The samples collected in 2018 were used for Illumina 16S and ITS amplicon sequencing.

### Characteristics of soil in different fertilization strategies

Soil organic matter (OM) content is one of the main issues of soils and agriculture in general. Total nitrogen (TN), total phosphorus (TP), total potassium (TK) were measured according to the Semi-micro Kjeldahl method, NaOH melting molybdenum antimony colorimetric method and the flame emission spectrometry method, respectively [15]. The details of OM, TN, TP and TK detection were performed by the steps described by Bao [16].

Soil aggregate stability is a measure of the ability of soil aggregates to resist degradation when exposed to external forces such as water erosion and wind erosion, shrinking and swelling processes, and tillage. The composition and stability of soil aggregate were measured by wet aggregate stability method [17]. The stability of soil aggregates was calculated according to the formulas reported by Liu et al [18].

Soil microbial biomass carbon (SMBC) and nitrogen (SMBN) are important indices of soil bio-fertility. SMBC and SMBN were measured using the fumigation-extraction method [19]. Soil organic carbon (SOC) content was measured in air-dried and ground samples with an elemental analyzer (Vario Max C/N). The soil microbial quotient (SMQ) was calculated from the equation: SMQ = SMBC/SOC. Soil enzyme activity is an important index of soil biological activity and soil fertility. To evaluate this important indicator, five enzymatic activities in soil were analyzed including invertase, protease, urease, acid phosphatase, and catalase. The enzyme activity was determined according to the methods described by Yang [20] and Geisseler [21]. The geometric mean of enzyme activities (GMea) was calculated according to the mean value of each enzyme activity via GMea = $\sqrt[{5}]{Inv\times \mathrm{Pr}o\times Ure\times AcP\times Cat}$.

### Illumina 16S and ITS amplicon sequencing

For microbial genomic DNA extraction, a MOBIO PowerSoil DNA Isolation Kit (QIAGEN, USA) was used. Agarose gel electrophoresis was adopted as a rough measurement to assess the qualities of DNA. Genomic DNA was used as a template in PCR amplifications of the V3 and V4 region of the bacterial 16S rRNA gene, using the universal primers 338F (5’-3’: ACTCCTACGGGAGGCAGCAG) and 806R (5’-3’: GGACTACHVGGGTWTCTAAT).

As for the microbial community analysis, we used four pairs of primers to perform DNA amplification to study the diversity and abundance changes of Bacteria (338F, 5’-3’: ACTCCTACGGGAGGCAGCAG/806R, 5’-3’: GGACTACHVGGGTWTCTAAT), fungi (ITS1F, 5’-3’: CTTGGTCATTTAGAGGAAGTAA/ITS2R, 5’-3’: GCTGCGTTCTTCATCGATGC), nitrogen-fixing bacteria (nifHF, 5’-3’: AAAGGYGGWATCGGYAARTCCACCAC/nifHR, 5’-3’:TTGTTSGCSGCRTACATSGCCATCAT) and denitrifies bacteria (cd3aF, 5’-3’: GTSAACGTSAAGGARACSGG/R3cdR, 5’-3’:GASTTCGGRTGSGTCTTGA), respectively. PCR products were detected by Nanodrop (Thermo, USA). In particular, PCR products concentrations had to be adjusted to assure an even amplification in the later sequence libraries construction. Axygen Gel Extraction Kit (Axygen, USA) was used to collect the target fragments of DNA. The densities of the collected fragments were detected by Qubit2.0 (Life Tech, USA) and quality control was performed with Agilent 2100 Bioanalyzer (Agilent, USA). Quantitative PCR (qPCR) was performed to test the efficiency of the adapters. Based on the efficiency, the clone libraries were diluted to a proper concentration for sequencing. Miseq system (Illumina, USA) were used to accomplish the sequencing under pair-end (PE) 300 bp mode.

### Sequence data analysis

Sequencing data was separated according to the Barcode and PCR primer sequences, which were then depleted. Data splicing and quality filtering were performed as usually using FLASH (v1.2.7; http://ccb.jhu.edu/software/FLASH/), Qiime (v1.9.1; http://qiime.org/scripts/split_libraries_fastq.html) and UCHIME algorithm (http://www.drive5.com/usearch/manual/uchime_algo.html), respectively. Operational taxonomic units (OTUs) clustering conducted using Uparse software (version 7.0.1001; http://drive5.com/uparse/) based on the threshold of 97% identity. The abundance (reads number) of OTUs in each sample was calculated, and OTUs with more than two reads were used for further analysis. The alpha diversity indicators (Chao1, ACE, observed OTUs, Shannon and Simpson) of the sequencing data within each group (n = 4) and beta diversity index (Unweighted UniFrac distance) of each sample was calculated. Principal Co-ordinates Analysis (PCoA) of samples was performed based on the Unweighted UniFrac distance of beta diversity index. SILVA rRNA database (http://www.arb-silva.de/) on Mothur website (http://www.mothur.org/wiki/RDP_reference_files) was queried for the annotation of the OTUs. OTUs relative abundances was calculated, and taxonomy assignment was performed using Ribosomal Database Project (RDP) classifier (80% confidence).

### Statistical analyses

For soil properties data, statistical analysis was performed following previous studies [22, 23]. GraphPad Prism 8 software was used for data statistical analysis. The soil properties data were expressed as the mean ± SD and analyzed by one-way ANOVA followed by multiple comparison with Tukey test. All levels of significance referred in the results is *p* < 0.05.

## Results

### Characteristics of soil in different fertilization strategies

**Fig 2:**
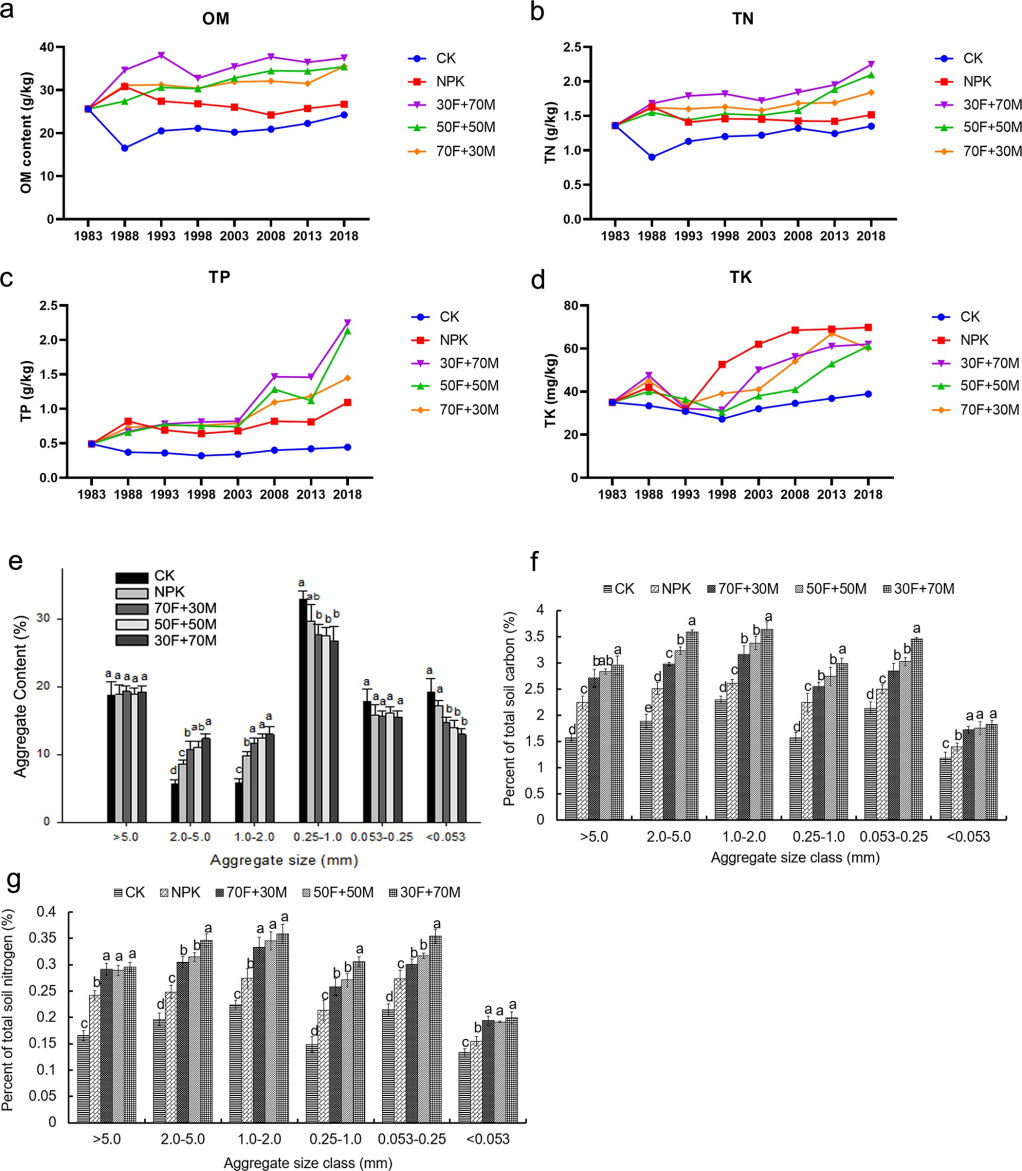
Impacts of various fertilizers on soil properties. (**a**) Dynamics of OM content from 1983 to 2012. (**b**) Proportion of water stable aggregates in different soil aggregate size class. (**c**) Percentage of total soil carbon in different soil aggregate size class. (**d**) Percentage of total soil nitrogen in different soil aggregate size class.

The long terms of different fertilization strategies significantly affected the OM content in soil. As expected, the higher the proportion of organic fertilizer was, the higher the soil OM content was (Fig 2A). In addition, the content of TN showed a slight increase or fluctuation in all the groups. And the higher the proportion of organic fertilizer was, the higher the soil TN content was (Fig 2B). For TP content, organic fertilizer treatment increased the content of TP (after 2003). But TP showed slight increase and no increase in NPK and CK, respectively (Fig 2C). The application of balanced chemical fertilizers and organic fertilizers could significantly increase the content of TK, and the effect of NPK was more obvious (Fig 2D).

Soil aggregate stability results showed that the content of water stable aggregates with a particle size of 0.25–1.0 mm was the highest and 2.0–5.0 mm particles was the lowest (Fig 2E). Long term application of organic fertilizer significantly increased the content of 1.0–2.0 mm and 2.0–5.0 mm particle content, but reduced particles of 0.25–1.0 mm and < 0.053 mm (Fig 2E). The contents of total soil carbon (TSC) and total soil nitrogen (TSN) in soil water stable aggregates increased with the increasing of the proportion of organic fertilizer application. In addition, the content of TSC and TSN in soil water stable aggregates of 1.0–2.0 mm were the highest in all treatments (Fig 2F and 2G). Table 1 showed the SMBC and SMBN content in different groups. As expected, 30F+70M group had the highest SMBC and SMBN value. Also, the mean value of the SMQ ranged from 1.63 to 3.24%, and the organic fertilizer group was higher than that of NPK and CK group.

**Table 1:** Changes of SMBC and SMBN content and SMQ in different groups.

Groups	SMBC (mg/kg)	SMBN (mg/kg)	SMQ (%)
CK	190.9±14.1 ^d^	28.3±3.4 ^d^	1.63±0.12 ^c^
NPK	396.0±29.2 ^c^	63.9±7.6 ^c^	2.61±0.32 ^b^
70F+30M	562.1±21.1 ^b^	89.6±6.9 ^b^	3.05±0.32 ^ab^
50F+50M	589.5±29.5 ^b^	99.5±7.1 ^ab^	3.10±0.22 ^a^
30F+70M	633.5±19.9 ^a^	104.6±5.6 ^a^	3.24±0.24 ^a^

CK: non-fertilization group; NPK: balanced inorganic fertilization; 70F+30M: 70% chemical and 30% organic fertilizer group; 50F+50M: 50% chemical and 50% organic fertilizer group; 30F+70M: 30% chemical and 70% organic fertilizer group; SMBC: Soil microbial biomass carbon; SMBN: Soil microbial biomass nitrogen. SMQ: The soil microbial quotient. For all variables with the same letter, the difference between the means is not statistically significant (*p* > 0.05). If two variables have different letters, they are significantly different (*p* < 0.05).

As for soil enzyme activity detection, invertase, protease, urease, acid phosphatase, and catalase had the highest and lowest activity in 30F+70M and CK group, respectively. GMea ranged from 4.19 to 9.38%, and the organic fertilizer group was significantly higher than that of NPK and CK group (Table 2).

**Table 2:** Soil enzyme activity of different fertilization strategies.

Groups	Invertase (mg/g/24 h)	Urease (mg/g/24 h)	Protease (μg/g/2 h)	Acid phosphatase (mg/g/h)	Catalase (ml/g/20 min)	GMea
CK	4.02±0.36 ^c^	0.47±0.05 ^d^	101.7±8.28 ^d^	0.26±0.04 ^c^	2.56±0.28 ^c^	4.19±0.19 ^d^
NPK	10.6±0.47 ^b^	0.73±0.05 ^c^	176.2±9.20 ^c^	0.38±0.06 ^b^	2.73±0.20 ^c^	6.80±0.12 ^c^
70F+30M	12.9±1.10 ^a^	0.88±0.01 ^b^	205.4±8.65 ^b^	0.59±0.06 ^a^	3.37±0.16 ^b^	8.73±0.31 ^b^
50F+50M	13.0±0.79 ^a^	0.88±0.05 ^b^	219.8±6.11 ^ab^	0.62±0.08 ^a^	3.72±0.24 ^ab^	9.05±0.11 ^ab^
30F+70M	14.5±1.43 ^a^	0.96±0.04 ^a^	225.9±15.3 ^a^	0.64±0.04 ^a^	3.88±0.24 ^a^	9.38±0.26 ^a^

CK: non-fertilization group; NPK: balanced inorganic fertilization; 70F+30M: 70% chemical and 30% organic fertilizer group; 50F+50M: 50% chemical and 50% organic fertilizer group; 30F+70M: 30% chemical and 70% organic fertilizer group; GMea: Geometric mean of enzyme activities. For all variables with the same letter, the difference between the means is not statistically significant (*p* > 0.05). If two variables have different letters, they are significantly different (*p* < 0.05).

### Illumina sequencing summary

In this study, four kinds of amplified fragments were studied including 16s (V3 and V4), *ITS2*, *nifH* and *nirS* genes of microorganisms in soil samples. After filtering out the low-quality reads and trimming the sequence adapter, we finally identified 867,874, 1,192,928, 276,062 and 373,121 high quality sequences from the 20 soil samples in the amplified fragments of 16s (V3 and V4), *ITS2*, *nifH* and *nirS*, respectively (S1 Table). In order to evaluate whether the amount of data is enough, rarefaction curve and rank abundance curve were constructed. The dissolution curve results (based on coverage index) showed that with the increase of extracted data, the number of OTUs tend to be gently (S1A–S1D Fig). The higher species rank value of samples showed that most of species range from 2,000–3,500 in bacteria, 300–600 in fungi, 500–800 in nitrogen-fixing bacteria and 200–400 denitrifies bacteria (S1E–S1H Fig). In addition, the PCA results revealed the excellent repeatability of all the test samples (S1I–S1L Fig). All the basic statistical results indicated the excellent sequence data quality and consistency of repetitions.

### Effects of different fertilization strategies on bacterial community

Compared with CK group, fertilization significantly improved the bacterial diversity, and the combination of chemical and organic fertilizer was more advantageous than chemical fertilizer only (Table 3). Group 70F+30M had the highest shannon index (7.09±0.05) and NPK had the lowest (6.89±0.05). As for ace, the trend is similar to Shannon, 70F+30M had the highest ace value (4061±109.93) and NPK had the lowest (3707±83.98).

**Table 3:** Alpha diversity index of bacteria in different groups.

Groups	shannon	simpson	ace	chao	coverage
CK	6.97±0.09 ^ab^	0.0027±0.0007 ^ab^	3967.29±149.72 ^a^	3987.60±145.37 ^a^	0.9710±0.0064 ^ab^
NPK	6.89±0.05 ^b^	0.0025±0.0002 ^b^	3707.23±83.98 ^b^	3720.01±85.01 ^b^	0.9660±0.0018 ^b^
70F+30M	7.09±0.05 ^a^	0.0020±0.0002 ^c^	4061.90±109.93 ^a^	4055.13±95.65 ^a^	0.9702±0.0020 ^a^
50F+50M	7.07±0.06 ^ab^	0.0021±0.0003 ^bc^	3986.80±59.33 ^a^	3972.70±75.32 ^a^	0.9650±0.0015 ^b^
30F+70M	7.07±0.08 ^ab^	0.0020±0.0004 ^bc^	3806.47±111.06 ^b^	3809.24±107.48 ^a^	0.9685±0.0021 ^ab^

CK: non-fertilization group; NPK: balanced inorganic fertilization; 70F+30M: 70% chemical and 30% organic fertilizer group; 50F+50M: 50% chemical and 50% organic fertilizer group; 30F+70M: 30% chemical and 70% organic fertilizer group; For all variables with the same letter, the difference between the means is not statistically significant (p > 0.05). If two variables have different letters, they are significantly different (p < 0.05).

**Fig 3:**
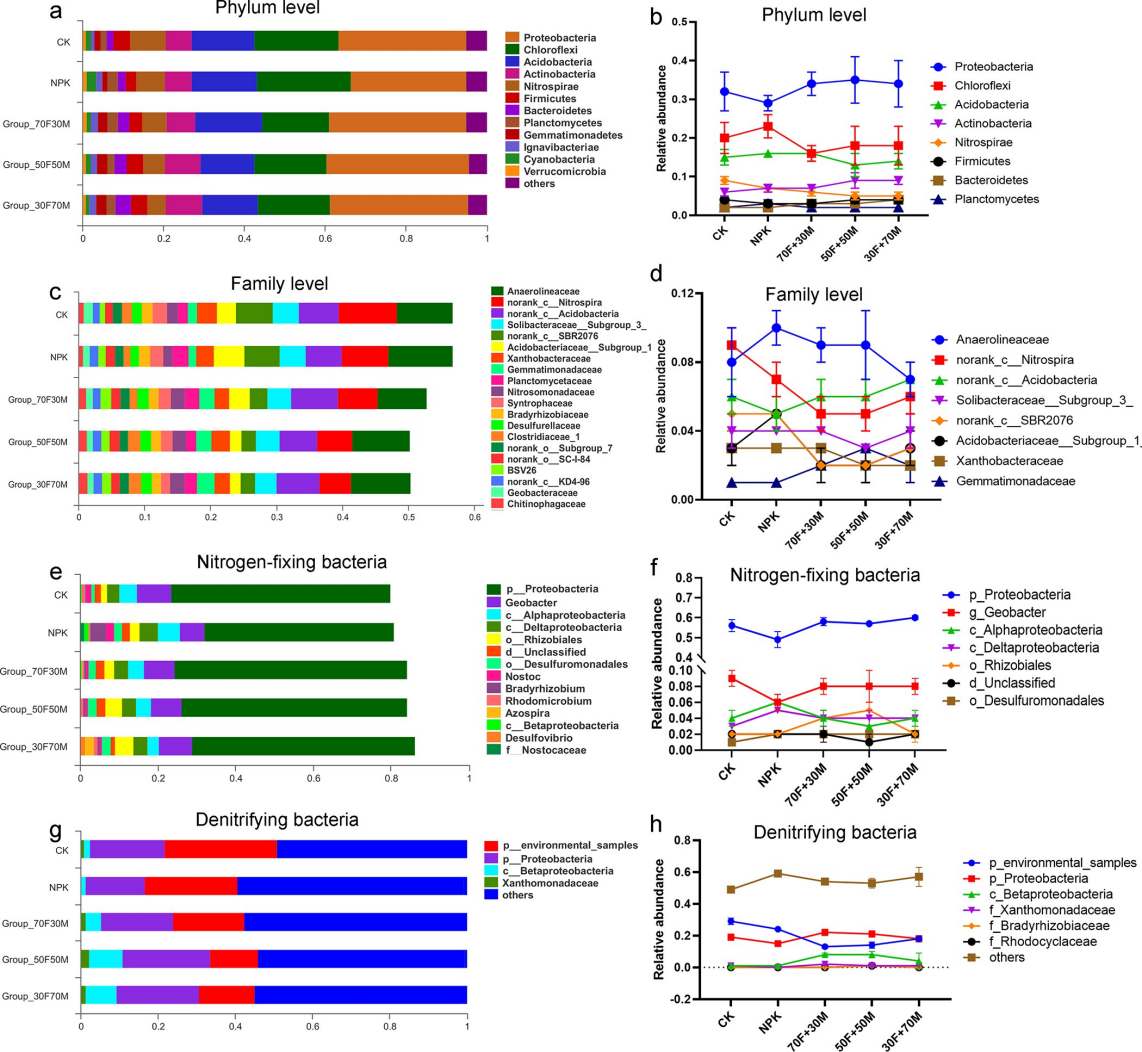
Impacts of various fertilizers on bacterial community. (**a**) Proportion changes in phylum level. (**b**) Relative abundance in phylum level. (**c**) Proportion changes in family level. (**d**) Relative abundance in family level. (**e**) Proportion changes of nitrogen-fixing bacteria. (**f**) Relative abundance of nitrogen-fixing bacteria. (**g**) Proportion changes of denitrifying bacteria. (**h**) Relative abundance of denitrifying bacteria.

The 16S rRNA sequence results showed that the composition of the bacteria was changed after fertilization, especially in the groups of combining chemical and organic fertilizer. At phylum level, the application of organic fertilizer reduced the relative abundance of *Chloroflexi*, *Acidobacteria* and *Nitrospirae*, but increased *Proteobacteria* and *Actinobacteria* (Fig 3A and 3B). At family level, *Anaerolineaceae*, *Nitrospira* (class), *Acidobacteria* (class), *Solibacteraceae_Subgroup_3* and *SBR2076* (class) were the dominate bacteria (Fig 3C). *Nitrospira* (class) and *SBR2076* (class) had a higher relative abundance in CK group, but declined sharply with application of fertilization, especially under organic fertilizer treatment (Fig 3C and 3D). In addition, Proteobacteria (phylum), *Geobacter* (genus), *Rhizobiales* (order) and *Alphaproteobacteria* (class) were the dominate nitrogen-fixing bacteria (Fig 3E). The relative abundance of Proteobacteria (phylum) was over 50%, which decreased in NPK but increased in organic fertilizer groups (Fig 3F). As for denitrifying bacteria, *Betaproteobacteria* (class) was significantly increased by organic fertilizer (Fig 3G and 3H). The application of organic fertilizer had a great influence on the soil bacteria community, and some bacteria were positively affected by proportion of organic fertilizer, such as *Proteobacteria* and *Actinobacteria*; some were negatively affected by organic fertilizer, such as Chloroflexi, Nitrospirae and Acidobacteria phylum.

### Effects of different fertilization strategies on fungal community

**Fig 4:**
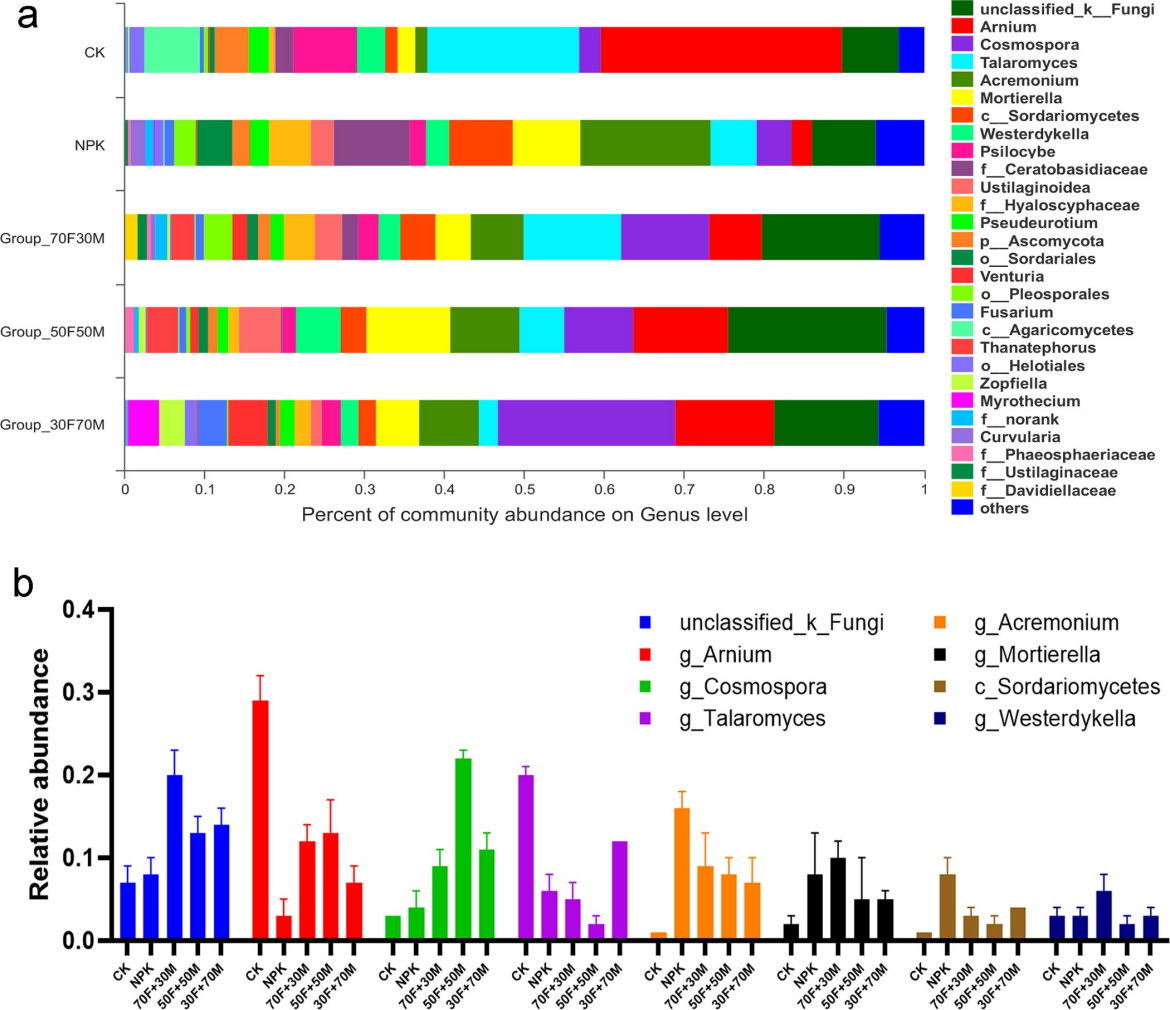
Impacts of various fertilizers on fungal community. (a) Proportion changes in genus level. (b) Relative abundance of fungal genus. Different colors represent different fungi genera. CK: non-fertilization group; NPK: balanced inorganic fertilization; 70F+30M: 70% chemical and 30% organic fertilizer group; 50F+50M: 50% chemical and 50% organic fertilizer group; 30F+70M: 30% chemical and 70% organic fertilizer group.

Alpha diversity index statistical analysis showed that Shannon, simpson, ace and chao were significantly different among different groups (Table 4). All the alpha diversity indexes revealed that NPK had more fungal than that of CK. Shannon, ace and chao 1 in combination of chemical and organic fertilizer groups were significantly higher than that in CK. In addition, there were no significantly difference between organic fertilizer groups and non-organic fertilizer group (NPK). Chemical fertilizer decreased the abundance of *Arnium*, of which the decline was alleviated with the addition of organic fertilizer (Fig 4A and 4B). For *Cosmospora*, the relative abundance increased after fertilization, in particular the combination of chemical and organic fertilizer. The relative abundance of *Acremonium* and *Mortierella* were also greatly increased by different fertilization strategies. However, when high proportion of organic fertilizer was applied, the abundance of *Acremonium* and *Mortierella* decreased.

**Table 4:** Alpha diversity index of fungi in different groups.

Groups	shannon	simpson	ace	chao	coverage
CK	2.96±0.09	0.13±0.02	602.73±31.26	609.78±38.47	0.9976±0.0003
NPK	3.47±0.14	0.07±0.01	678.30±42.36	657.90±49.49	0.9978±0.0003
70F+30M	3.20±0.32	0.10±0.05	751.15±27.19	652.66±33.43	0.9972±0.0003
50F+50M	3.49±0.11	0.06±0.01	770.04±99.45	722.11±55.76	0.9973±0.0004
30F+70M	3.26±0.18	0.10±0.03	649.06±34.99	673.96±29.85	0.9974±0.0003

CK: non-fertilization group; NPK: balanced inorganic fertilization; 70F+30M: 70% chemical and 30% organic fertilizer group; 50F+50M: 50% chemical and 50% organic fertilizer group; 30F+70M: 30% chemical and 70% organic fertilizer group; For all variables with the same letter, the difference between the means is not statistically significant (*p* > 0.05). If two variables have different letters, they are significantly different (*p* < 0.05).

## Discussion

Because of long-term weathering and nutrient leaching, the fertility of ORC is very low [24]. In order to change this situation, fertilization is very necessary. OM content is one of the important indicators of soil quality. In our results, the application of chemical fertilizer could promote the content of OM, but the combination of organic fertilizer can play a stronger role. Also, there are many studies reported that organic fertilizer could improve OM content [25, 26]. We found that the OM content significantly increased with the increasing percentage of organic fertilizer, which indicated the important role of organic fertilizer in the accumulation of soil OM. Soil stable stability is important soil property that contributes to the maintenance of a porous soil structure and associated water movement [27]. Also, soil stable stability is a good indicator of a soil’s susceptibility to erosion. Stable soil aggregates play a key role in soil quality, since they protect organic material from microbial decomposition [28]. In our results, different fertilization strategies had significant interactive effects on soil stable stability. Fertilized soil had larger stable stability aggregates, and the application of organic fertilizer made the particle aggregation more obvious. The application of organic fertilizer mediated the increase of 1–5 mm aggregates ratio and the decrease of aggregates less than 1mm (Fig 2E). This may suggest that the addition of organic fertilizer is important in soil aggregation. As for SMBC and SMBN, the contents of which increased along with the increased proportion of organic fertilizer (Fig 2F and 2G). This could be due to organic matter decomposing and providing a large amount of available substrates for soil microbial growth [29].

Yu had reported that soil microbial biomass could be sensible to indicate the soil quality of the degraded red soil [30]. Therefore, the abundance and diversity of microorganisms in soil are extremely important for soil. Fertilization could reduce the diversity index, richness index and evenness index of bacterial community, and chemical fertilizer treatment decreased the most. While in groups containing organic fertilizer, indexes of bacterial community increased with the increase of the proportion of organic fertilizer, which showed that the combination of organic and inorganic fertilizer was beneficial to the stability of ORC bacterial community diversity. Interestingly, inorganic fertilizer was also showed stabilizing effects on fungi community. Previous studies had reported that application of organic fertilizers produced changes in soil physicochemical parameters and soil respiration and nutrient cycling activities, which were often associated with differences in soil bacterial community structure and diversity [31–33]. This theory was also applicable to the improvement of ORC fertility. This 30-year study also show that long-term combination of organic and inorganic fertilization can effectively stabilize ORC microbial biomass and fertility.

Long-term fertilization had great influence on microbial structure. In our results, *Proteobacteria* was the most abundant phylum, which was similar with Eo’s report [34]. The increased abundance of *Proteobacteria* by organic fertilizer indicated that organic fertilizer could provide a more suitable living environment for it, which was beneficial to increase soil microbial biomass. *Chloroflexi* is another dominate phylum. Lopez found the predominance of *Chloroflexi* in soil, but decreased by the addition of nickel [35]. *Chloroflexi* affiliated bacteria are the primary degraders of polysaccharides in the anoxic zones of rice field soils [36]. In addition, Aileen found that the bacterial communities were dominated by *Proteobacteria* and *Chloroflexi*, which was decreased after organic fertilizer application [32], which was highly consistent with our results. We also found that, the abundance of nitrogen-fixing bacteria under Proteobacteria phylum and *Rhizobiales* order were increased, which demonstrated that organic fertilizer could improve the living conditions for nitrogen-fixing bacteria. As for fungi, the diversity indexes were increased by fertilization, which revealed the important role of organic fertilizer for fungi. Fungi such as *Arnium*, *Agaricomycetes* and *Talaromyces* were decreased comprised with CK. *Talaromyces* is distributed widely in soil, debris, organic matters and marine invertebrates [37, 38], which play a role in rice disease resistance [39]. The decrease of *Talaromyces* is likely due to the complex interaction of organic fertilizer in the soil. Meanwhile, organic fertilization might also affect the disease resistance of rice through *Talaromyces*. In our results, *Cosmospora* was positively regulated by fertilization, and in particular organic fertilizer. Although the role of *Cosmospora* in soil was not clear, the changes reflected the important role of organic fertilizer.

## Conclusion

Long term application of organic fertilizer significantly increased the organic matter (OM) content, which result in the increase of total carbon and nitrogen in soil. Bacteria such as *Proteobacteria*, *Chloroflexi* and some nitrogen-fixing bacteria were increased by the combination of high proportion of organic and low proportion of inorganic fertilizer. Fungi such as *Arnium*, *Agaricomycetes* and *Talaromyces* were decreased in fertilization groups comprised with CK. While some fungi, such as *Talaromyces*, were limited by the complex interaction of organic fertilizer in the soil.

## Supporting information

S1 Fig Rank–abundance distribution curves. (**a-d**) Coverage curves of 16s V3&V4, nifH, nirS and fungi ITS genes. (**e**-**h**) Rank–abundance distribution curves of 16s V3&V4, nifH, nirS and fungi ITS. (**i-l**) Principal component analysis of 16s V3&V4, nifH, nirS and fungi ITS genes.
(TIF)

S1 Table Summary of NGS sequencing data. (XLSX)
