# Supplementary figures and images for: Long-term effects of combination of organic and inorganic fertilizer on soil properties and microorganisms in a Quaternary Red Clay
Source: PLoS One. 2021 Dec 16;16(12):e0261387. doi: 10.1371/journal.pone.0261387 (PMC8675731; doi:10.1371/journal.pone.0261387)

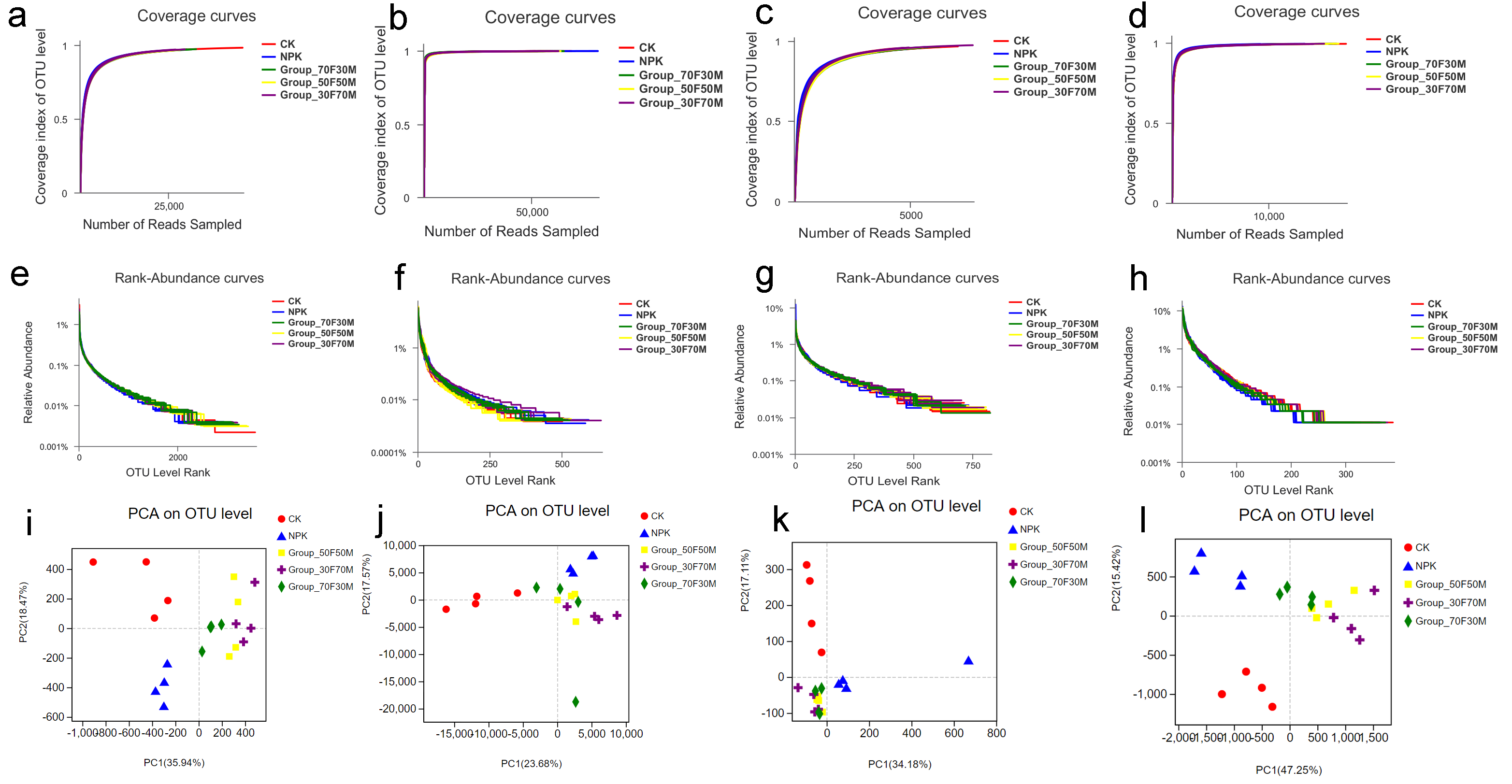

Supplement: S1 Fig — (a-d) Coverage curves of 16s V3&V4, nifH, nirS and fungi ITS genes. (e-h) Rank–abundance distribution curves of 16s V3&V4, nifH, nirS and fungi ITS. (i-l) Principal component analysis of 16s V3&V4, nifH, nirS and fungi ITS genes. (TIF) [file pone.0261387.s001.tif]
